# Nociceptive Pathway in the Cockroach *Periplaneta americana*

**DOI:** 10.3389/fphys.2019.01100

**Published:** 2019-08-21

**Authors:** Stav Emanuel, Frederic Libersat

**Affiliations:** Department of Life Sciences and Zlotowski Center for Neurosciences, Ben Gurion University, Beer Sheva, Israel

**Keywords:** nociception, insect, interneurons, nocifensive behavior, nociceptive receptor, extracellular recording

## Abstract

Detecting and avoiding environmental threats such as those with a potential for injury is of crucial importance for an animal’s survival. In this work, we examine the nociceptive pathway in an insect, the cockroach *Periplaneta americana*, from detection of noxious stimuli to nocifensive behavior. We show that noxious stimuli applied to the cuticle of cockroaches evoke responses in sensory axons that are distinct from tactile sensory axons in the sensory afferent nerve. We also reveal differences in the evoked response of post-synaptic projection interneurons in the nerve cord to tactile versus noxious stimuli. Noxious stimuli are encoded in the cockroach nerve cord by fibers of diameter different from that of tactile and wind sensitive fibers with a slower conduction velocity of 2–3 m/s. Furthermore, recording from the neck-connectives show that the nociceptive information reaches the head ganglia. Removing the head ganglia results in a drastic decrease in the nocifensive response indicating that the head ganglia and the nerve cord are both involved in processing noxious stimuli.

## Introduction

An animal’s ability to detect environmental threats, which may damage an animal’s body, is defined as nociception. Nociception consists of the physiological transduction of stimuli that impair the integrity of the tegument of an animal, the processing of such sensory information, and an adaptive motor response to this sensory input. Here, we define a nocifensive behavior as a defensive behavior that is elicited by sensory stimuli that have the potential to cause injury. Because of its strong adaptive benefit, nociception is common to most animal phyla and is thought to rely on similar cellular and physiological processes (Smith and Lewin, 2009). Among invertebrates, nociception as a system has been investigated mostly in Mollusks and Arthropods (Smith and Lewin, 2009; Burrell, 2017). With regard to pain, the question as to whether there is an additional system associated with nociception that encodes the unpleasantness (suffering) is highly debated (Elwood, 2011; Sneddon, 2015; Walters, 2016). Insects, the largest and most studied group within the arthropod phylum, are known to respond to noxious stimuli with escape like behaviors that can be considered as nocifensive behaviors (Eisemann et al., 1984; Keller, 2017; Pali-Schöll et al., 2018). For example, *Manduca sexta* larvae show nocifensive behavior expressed by a rapid bending response to sharp poking or pinching stimuli applied to all abdominal segments (Walters et al., 2001). There has been a recent renewed interest in insect nociception using Drosophila as a model. For example, Drosophila larvae and adults produce a stereotyped defensive behavior in response to noxious mechanical, chemical or thermal stimuli (Tracey et al., 2003; Xu et al., 2006; Fiala, 2008; Neely et al., 2011; Im and Galko, 2012; Honjo et al., 2016; Tokusumi et al., 2017; Hu et al., 2018). This type of behavior is also evoked in cockroaches in response to thermal noxious stimuli (Gritsai et al., 2004; Gritsai et al., 2009; Maliszewska et al., 2018). With this mind, the present paper has examined the nociceptive pathway from sensory reception to motor behavior in the cockroach *Periplaneta americana* using primarily electrophysiological recordings. Here, we will restrict our study to the physiological pathway involved in the ability of an animal to detect and react to noxious stimuli that could potentially impair its tegument by escaping away from that stimulus.

## Materials and Methods

### Animals

Adult male cockroaches (*P. americana*) were raised in crowded conditions in plastic containers (50 × 50 × 70 cm) under a 12D:12L cycle at 26°C. Water and food (cat chow) were provided *ad libitum*.

### Calibrated Stimuli

We used a custom-built device developed in our lab to apply calibrated tactile and noxious stimuli. The device is based on a step motor that drives a metal tip with changing temperatures mounted on a micromanipulator. Briefly, it consists of the head of a miniature cauterizer mounted on a rod attached to a step motor. The heat of the probe (100°C) and the movement of the step motor are remotely controlled by an Arduino board and amplified using a 5 V, 1.2 A power source and a ULN2003. Using this device, tactile or combination of tactile and noxious heat stimuli (0.5 s or 3 s) were applied (referred to as “brief” stimuli). The noxious (heat) stimulus temperature was adjusted to not result in any damage of the cuticle. The response of tactile sensory neurons is phasic and lasts at the most 20 ms undergoing rapid adaptation (French, 1984; Pollack et al., 1995). The response of tactile interneurons is also phasic and lasts at the most 40 ms undergoing rapid adaptation (Ritzmann and Pollack, 1994). Moreover, tactile response does not change with increasing stimulus duration (Ritzmann and Pollack, 1994). Hence, tactile units were evoked only at the onset and offset of the brief noxious stimulus. In addition, to distinguish between the tactile and noxious components of the stimulus the “cold” probe was placed on the cuticle for at least 10 s and, without removing the probe, it was heated by turning on the heat switch on the Arduino board (referred to as “continuous” stimuli). For this stimulus, tactile units were rarely evoked when the noxious heat stimulus was activated.

### Behavior

Measurements of behavioral response of cockroaches to noxious stimuli were made in tethered cockroaches standing on a slippery glass platform covered with mineral oil. A photo-resistor was placed under the hind leg while a light source was placed above the same leg to monitor leg movement (as described in Fouad et al., 1994) which indicates walking or escape. Each recorded spike from the photo-resistor corresponds to a single leg step. Noxious or tactile stimuli (0.5 s) or continuous (transition from tactile to noxious stimuli) stimuli were applied to the abdomen. This set up was also used to test the involvement of the cockroach’s head ganglia in nocifensive behavior. In this behavioral assay, the cockroach head was surgically removed under cold anesthesia.

### Electrophysiology

**FIGURE 1:**
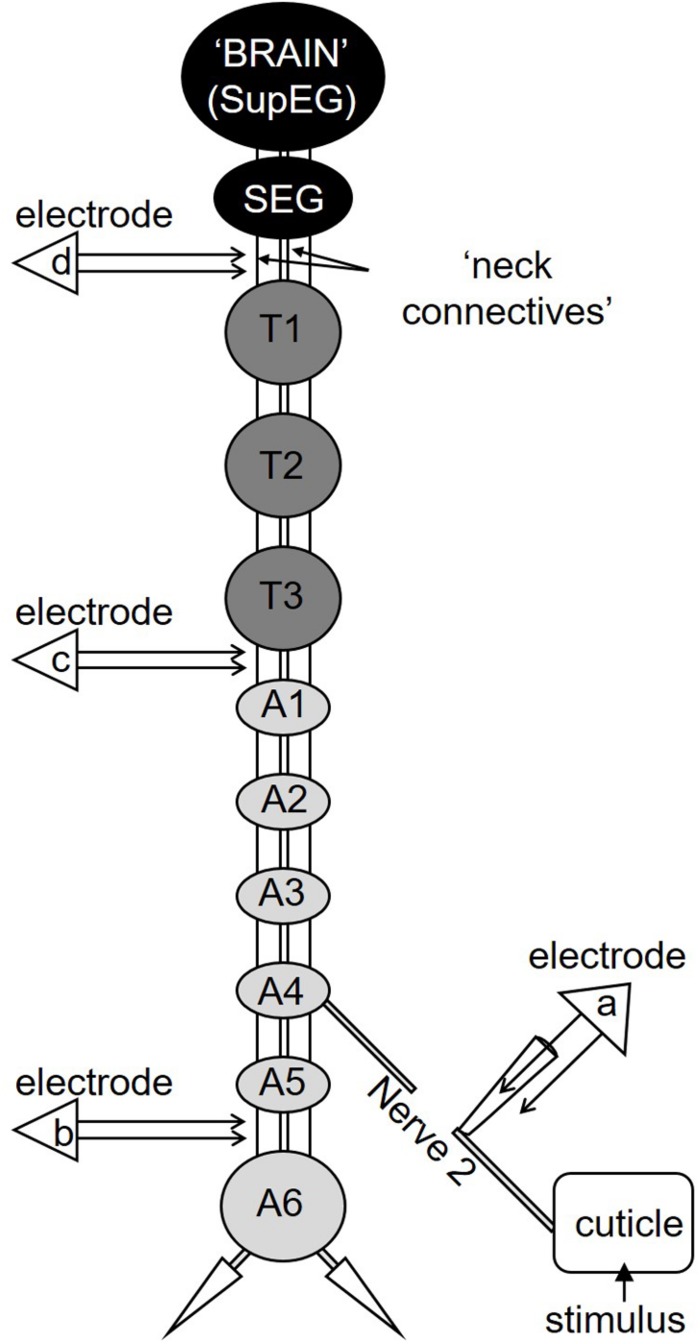
A diagram depicting the cockroach’s nervous system. The cockroach’s nervous system comprises two head ganglia (the SupEG or “Brain” and the SEG; black) three thoracic ganglia (T1–T3; dark gray) and six abdominal ganglia (A1–A6; light gray). Two connectives link between adjacent ganglia. The connectives between the head and thorax are termed “neck connectives.” One example of the peripheral Nerve 2 is illustrated on one side of the abdominal ganglion A4. In this preparation Nerve 2 is severed and suction electrode “a” is positioned so the recording is of the sensory input from the cuticle only. Electrodes “b,” “c,” and “d” are shown in the appropriate location along the nervous system and are placed on a single connective.

The preparation has already been described (Libersat et al., 1989). Briefly, the cockroach was anesthetized with CO_2_ and pinned dorsal side up on a recording platform after cutting the legs at the coxal-trochanter joint and the wings to stumps. The dorsal abdominal cuticle was removed and the nervous system was exposed from the third thoracic ganglion (T3) to the most posterior ganglion A6 (Figure 1). To record the axons of nociceptive sensory neurons, we used extracellular suction electrode recording of abdominal nerve 2 (Figure 1; electrode “a”). Nerve 2 and its branches is a mixed nerve with sensory and motor axons (Shankland, 1965). The nerve was severed between the recording site and the abdominal ganglion (A3 or A4) to record only the response of the sensory axons. Brief (3 s of either tactile or noxious stimulus) or continuous (transition from tactile to noxious stimuli) stimuli were given to the body segment innervated by the recorded abdominal nerve.

To establish if nociceptive information is carried along the cockroach’s central nervous system, extracellular recordings of projection interneurons from a single connective in the abdominal nerve cord were made using a silver bipolar hook electrode (Figure 1, electrode “b”). The connective on the recording side was crushed anterior to the recording site to record pure ascending activity. The contralateral connective was left intact. Brief (3 s) tactile or noxious stimuli or continuous (transition from tactile to noxious stimuli) stimuli were applied to the ninth abdominal body segment in the middle of the dorsal tergum. We confirmed that these stimuli recruit interneurons axons in both connectives (data not shown) and enabled us to measure a motor response. The response of the coxal depressor muscle of the hind leg was recorded with EMG nichrome fine wires electrodes. In most recordings, we could discriminate between the large amplitude EMG spike from the fast motor neuron (Df) and the smaller amplitude EMG spike of the slow motor neuron (Ds).

To measure the conduction velocity of interneurons which respond to noxious stimuli, two silver bipolar hook electrodes were positioned on the same connective along the cockroach’s nervous system (Figure 1, electrode “b” and “c”). The connective was crushed anterior to the most anterior recording site (between T3 and A1, see Figure 1) to remove descending activity. First, wind stimuli (brief wind puffs) were applied to the cerci to recruit the Giant interneurons. Then, continuous (transition from tactile to noxious stimuli) stimuli were applied to the ninth abdominal body segment in the middle of the dorsal tergum to recruit first the tactile interneurons and then the nociceptive interneurons. The time difference between two identified spikes from each electrode was measured and the distance between the two electrodes was measured after the recording. To ensure that our measurements were consistent with the conduction velocity of known interneurons axons in the nerve cord, we also measured the conduction velocity of the wind sensitive ventral and dorsal giant interneurons, termed vGIs and dGIs, respectively (Roeder, 1948; Spira et al., 1969; Westin et al., 1977).

To establish if nociceptive information reaches the cockroach’s head ganglia, we recorded from one of the neck connectives using a silver bipolar hook electrode (Figure 1, electrode “d”). The connective was crushed anterior to the recording site (below the subesophageal ganglion) to eliminate any activity/response descending from the head ganglia. Brief (0.5 s) or continuous (transition from tactile to noxious stimuli) stimuli were given to the last abdominal segment.

For all recording, a ground electrode (100 μm silver wire) was inserted in the metathoracic segment.

All electrical activity was recorded with an A-M Systems Model 1700 Differential AC Amplifier and sampled at 20 kHz using a CED Micro 1401 analog-to-digital board (Cambridge Electronic Design). The acquired activity was analyzed offline using Spike2 version 5.05 software (Cambridge Electronic Design) and then exported to Excel.

### Analysis and Statistics

The “escape duration” in behavioral tests and the time difference in conduction velocity tests was measured from the raw data. To measure the strength of response in extracellular recording a root mean square (RMS) procedure was applied to the waveform data. RMS is calculated by summing the square of each data point, dividing the sum by the number of data points and then taking the square root of the result. Then the area beneath the resulting waveform data was measured (“RMS Area”). To normalize the response strength, RMS area measurements before the stimulus was subtracted from the RMS area measurements after the stimulus. This technique of analysis is often used for quantifying EMGs and less often to quantify extracellular recordings. Hence, we applied RMS analysis to be able to compare the power of the responses of the sensory nerve, the interneurons, and the muscles. We applied spike counting and RMS analysis to the sensory nerve recordings to ensure that they yield the same results.

All statistical tests were performed using SigmaPlot 13.0 software. If data were not normally distributed according to the Shapiro–Wilk test, the non-parametric statistical tests were used. The statistical significance was determined by using *T*-test, Mann–Whitney Rank Sum Test, Paired *t*-test and Wilcoxon Signed Rank Test. For the behavioral tests log transformation was applied to the values to normalize the data.

## Results

### Nocifensive Behavior

**FIGURE 2:**
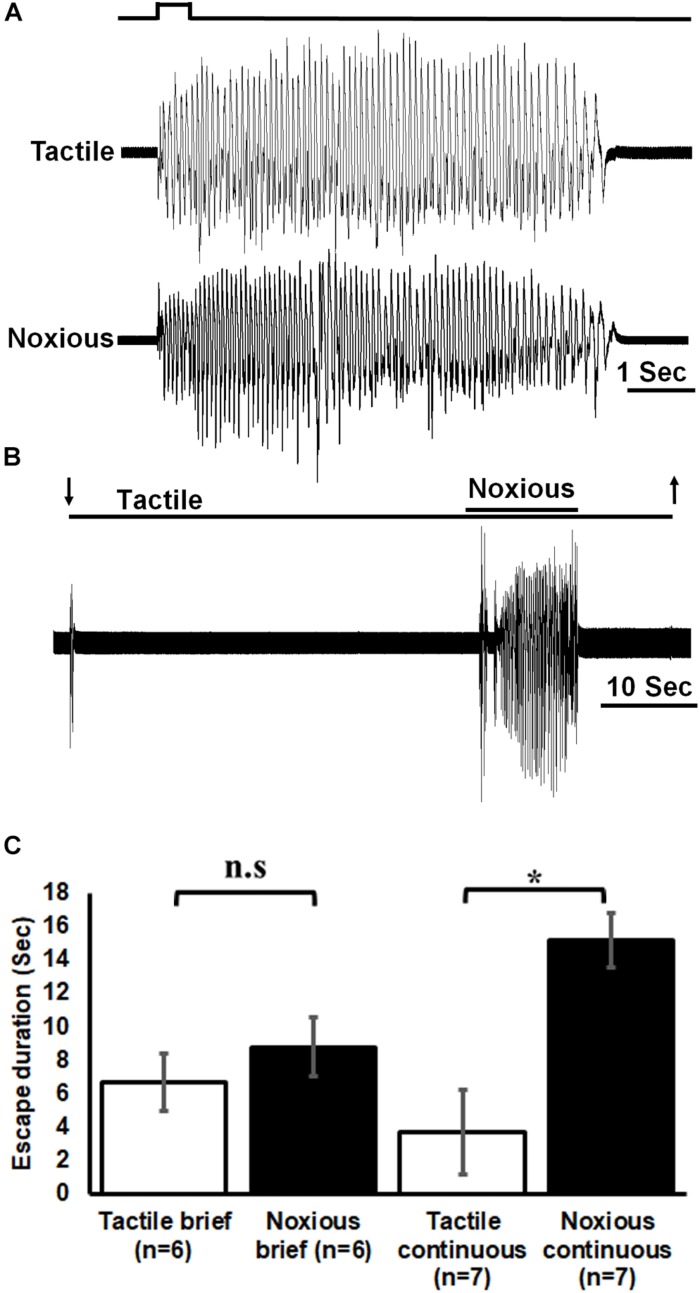
Noxious stimulus induces a nocifensive escape response. **(A)** Leg movements (which represent escape) of a cockroach in response to brief (0.5 s) tactile (upper trace) and noxious (lower trace) stimuli to the abdomen. Stimulus duration is indicated. **(B)** Leg movements in response to continuous tactile and noxious stimuli to the abdomen. Stimulus duration is indicated with horizontal lines and arrows (upper line: noxious; lower line: tactile). **(C)** Brief noxious and tactile stimuli display similar escape response. Continuous noxious stimuli induces a significantly higher escape duration than continuous tactile stimuli. Bars represent means ± SEM, significance is indicated with asterisk or with n.s.

We first tested the behavior of tethered but otherwise intact cockroaches to tactile and noxious stimuli. Brief (0.5 s) noxious and tactile stimuli evoked a fast running escape response in cockroaches on 27 trials for tactile and 28 trials for noxious for all cockroaches tested (*n* = 6; Figures 2A,C). The duration (in seconds) of roughly 5 escape responses was averaged for each cockroach. Then, the averages for all cockroaches were pooled and averaged. Escape response was similar for brief noxious and tactile stimuli (means ± SEM: noxious 8.8 ± 1.8, tactile 6.7 ± 1.7). However, since the brief noxious stimulus also included a tactile component that cannot be teased apart, we then used a pure continuous noxious stimulus as described in the methods. Such continuous noxious stimuli (*n* = 7, 16 trials, means ± SEM: 15.2 ± 1.6) induced a significantly longer (*P* < 0.05, Mann–Whitney rank sum test) escape duration than continuous tactile stimuli (*n* = 7, total 16 trials, means ± SEM: 3.7 ± 2.6; Figures 2B,C).

### Physiology of the Nociceptive Pathway

**FIGURE 3:**
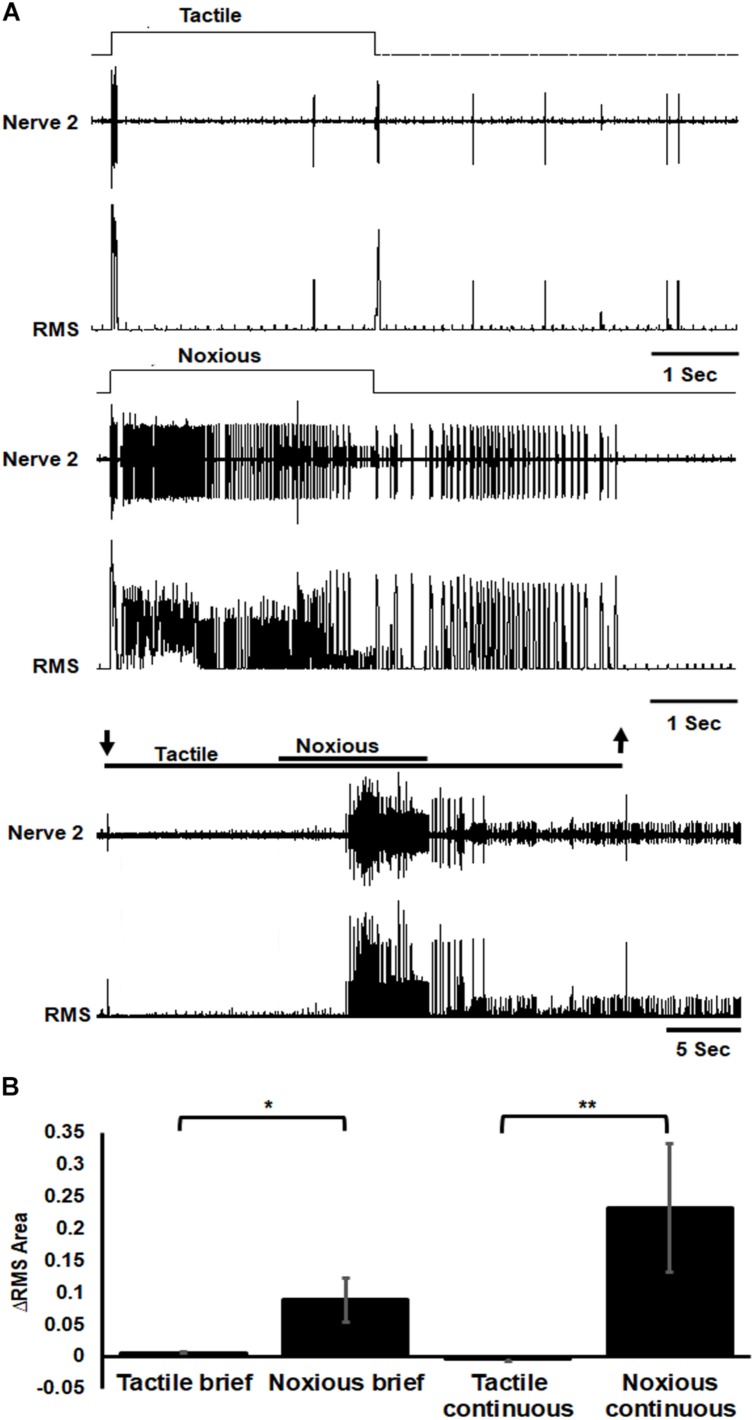
Sensory axons response to noxious stimulus is stronger than the response to tactile stimulus. **(A)** Extracellular recording of abdominal nerve 2 shows that the response to brief (3 s) tactile stimulus (two upper traces) is transient in the onset and offset of the stimulus while the response to brief (3 s) noxious stimulus (two middle traces) is prolonged and involves different units from that of tactile stimulus. Continuous transition from tactile to noxious stimuli (two lower traces) shows a transient response to tactile stimulus and an ongoing response to noxious stimulus. Stimulus duration is indicated with horizontal lines and arrows (upper line: noxious; lower line: tactile). The upper trace of each stimulus example (tactile, noxious or continuous transition) is the response of the nerve and the lower trace is the same data after RMS procedure. **(B)** The averaged RMS area shows that the response to brief or continuous noxious stimuli is stronger than that of tactile stimuli. Bars represent means ± SEM, significance is indicated with asterisk.

To investigate the nociceptive pathway underlying the nocifensive behavior, we started by characterizing the response of the sensory nociceptive neurons. The response of sensory axons in nerve 2 was different in response to tactile or noxious stimuli (Figure 3). The response to tactile stimulus was transient (phasic) to the onset and offset of the stimulus while the response to noxious stimulus was prolonged (phasic-tonic). Noxious stimuli recruited different units (sensory axons) from that recruited by the tactile stimuli (Figure 3A). The sensory response to noxious stimuli was also stronger for all types of stimuli (Figure 3B; brief tactile/noxious: *n* = 7 animals, *P* < 0.05, paired *t*-test; continuous tactile/noxious: *n* = 11 animals, *P* < 0.001, Wilcoxon signed rank test).

**FIGURE 4:**
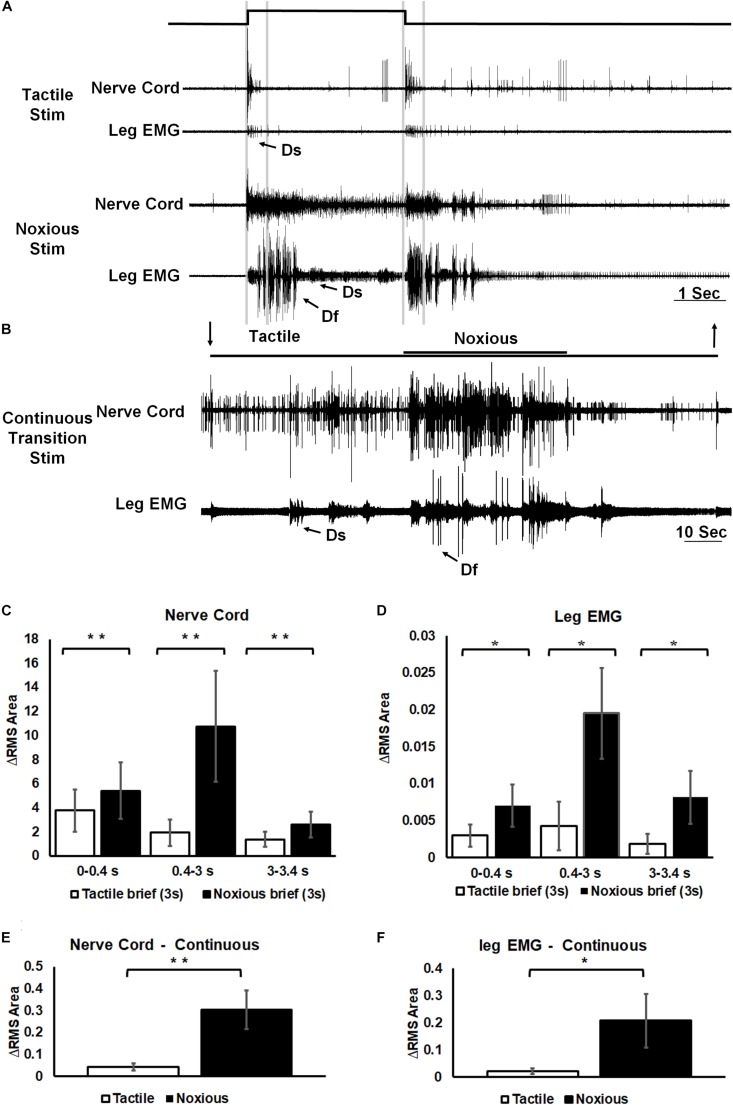
Noxious information is carried along the nerve cord and induces escape behavior. **(A)** Representative example of a simultaneous recordings from post-synaptic interneurons in the nerve cord (upper trace of each stimulus example) and the leg EMG (lower trace of each stimulus example) following brief (3 s) tactile (upper traces) and noxious (lower traces) stimuli. Vertical lines represent a time division (0–0.4, 0.4–3, and 3–3.4 s) corresponding to onset, duration and offset of the stimulus. Activity of the fast and slow motor neurons (Df and Ds, respectively) is indicated on the leg EMG traces. **(B)** Representative example of a simultaneous recordings from post-synaptic interneurons in the nerve cord (upper trace) and the leg EMG (lower trace) following a continuous transition between tactile and noxious stimuli. Stimulus duration is indicated with horizontal lines and arrows (upper line: noxious; lower line: tactile). Activity of Df and Ds is indicated on the leg EMG trace. **(C)** Averaged RMS area of nerve cord response to brief tactile and noxious stimuli, according to the mentioned time division. Panel **(D)** same as panel **(B)** for the leg EMG response. **(E)** Averaged RMS area of nerve cord response to a continuous transition between tactile and noxious stimuli. Panel **(F)** same as panel **(E)** for the leg EMG response. For panels **(C–F)** bars represent means ± SEM, significance is indicated with asterisk.

Similarly, the response of post-synaptic, inter-segmental projection interneurons in the nerve cord was different following tactile or noxious stimuli (Figure 4). Following a brief (3 s) tactile stimulus (Figure 4A, *n* = 20), large amplitude spikes from projections neurons were evoked at the beginning and the end of the stimulus. The response to a brief noxious stimulus (Figure 4A) consisted of a mixed response of tactile and nociceptive spikes followed by small amplitude spikes throughout the stimulus. The motor response measured with the EMG from the hind leg stump also showed qualitative and quantitative differences following brief tactile or noxious stimuli (Figure 4A, *n* = 13). In such tethered and dissected preparation, tactile stimuli evoked a response in the slow motor neuron (Ds) but rarely in the fast motor neuron (Df). In contrast, the tactile-noxious stimulus evoked a strong motor response with the recruitment of both Df and Ds. As in the preceding experiments on the sensory response, we also investigated the nociceptive component of the projection interneurons’ response by applying a continuous noxious stimulus. The nerve cord response to a continuous transition between tactile and noxious stimuli (Figure 4B, *n* = 13) started with a transient response to the tactile stimulus and an ongoing response to the noxious stimulus. The EMG response to this stimulus was stronger to the noxious stimulus and weaker to the tactile stimulus. Figures 4C,D show the difference in the response of the nerve cord and leg EMG, respectively, to brief stimuli by measuring RMS area. The response was binned in three time-segments corresponding to onset, duration and offset of the stimulus (0–0.4, 0.4–3, and 3–3.4 s; lines corresponding to this division shown in Figure 4A). A significant increase in response to noxious stimulus was found in all three time-bins of the stimulus for both nerve cord response (*P* < 0.001, paired *t*-test) and leg EMG response (*P* < 0.05, paired *t*-test). Figures 4E,F show the difference in the response of the nerve cord and leg EMG, respectively, to continuous transition stimuli by measuring RMS area. A significant increase in response to noxious stimulus was found for both nerve cord response (*P* < 0.001, paired *t*-test) and leg EMG response (*P* < 0.05, paired *t*-test).

**FIGURE 5:**
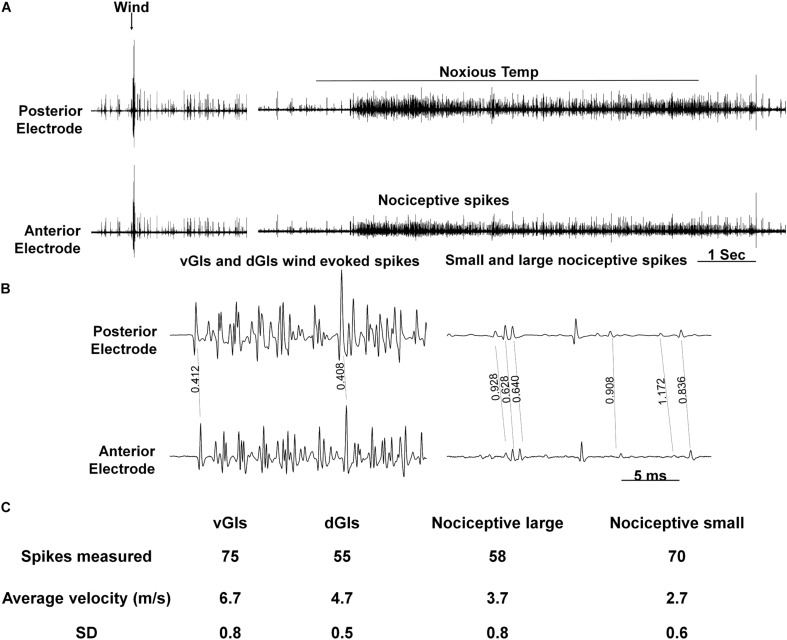
Conduction velocity of nociceptive projections neurons is two to three-fold slower than that of wind projection interneurons. **(A)** Response of interneurons in the nerve cord to a wind stimulus (left) and a continuous transition between tactile and noxious stimuli (right). Two electrodes were used to measure velocity (upper and lower traces represent posterior and anterior electrodes, respectively). **(B)** Enlarged time scale of the traces in panel **(A)**. Time differences (in seconds) between two similar spikes are shown. **(C)** A table summarizing the data from conduction velocity tests.

Next, to evaluate the conduction velocity of the nociceptive axons, we used two pairs of electrodes placed on both ends of the abdominal nerve cord (A5–A6 and T3–A1). The calculated conduction velocity of nociceptive projection interneurons was found to be different to that of wind sensitive projection interneurons (Figure 5, *n* = 6 animals). Based on the measurements of 70 spikes total (in all preparations) of the wind sensitive ventral giant interneurons (vGIs) which have the shortest response latency and highest spike amplitude, we calculated an average conduction velocity of 6.7 m/s (standard deviation or “SD” = 0.8). For the wind sensitive dorsal giant interneurons (dGIs), the average velocity based on 55 spikes total (in all preparations) was 4.7 m/s (SD = 0.5). The values measured in this work are similar to known values measured previously (Roeder, 1948; Spira et al., 1969; Westin et al., 1977) and confirm the validity of our method of measurements. For nociceptive projections interneurons, we observed two types of spikes amplitudes. Hence, we analyzed separately the conduction velocities of large amplitude versus smaller amplitude nociceptive spikes. 58 spikes of large amplitude had an average velocity of 3.7 m/s (SD = 0.8) and 70 spikes of small amplitude had an average velocity of 2.7 m/s (SD = 0.6). Therefore, the conduction velocity of these nociceptive projection interneurons is slower than that of wind sensitive interneurons.

**FIGURE 6:**
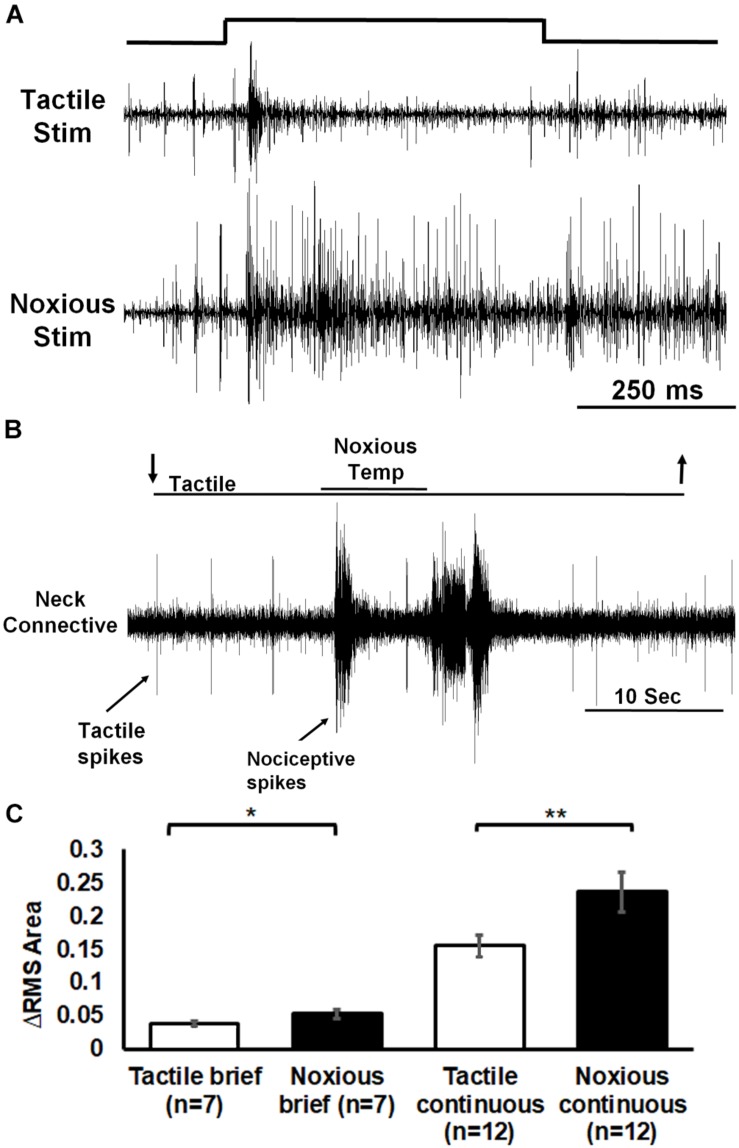
Extracellular recording of neck connective ascending activity following tactile and noxious stimuli shows that noxious information is carried along the nerve cord to the head ganglia. **(A)** Representative example of neck connective response to brief (0.5 s) tactile (upper trace) or noxious (lower trace) stimuli (stimulus duration is indicated). **(B)** Activity in the neck connective in response to a continuous transition between tactile and noxious stimuli. Stimulus duration is indicated with horizontal lines and arrows (upper line: noxious; lower line: tactile). **(C)** The averaged RMS area of the neck connective response to brief (0.5 s)/continuous noxious stimuli is significantly higher than that of brief/continuous tactile stimuli. Bars represent means ± SEM, significance is indicated with asterisk.

To explore the possibility that the head ganglia are involved in the processing nociceptive information, we first checked if such information reaches the head ganglia (Figure 6). Extracellular recording of ascending activity from a neck connective which links the thoracic to the head ganglia showed different responses to tactile and noxious stimuli. We first used brief (0.5 s) tactile (*n* = 7, means ± SEM: 0.04 ± 0.003) or noxious (*n* = 7, means ± SEM: 0.05 ± 0.01) stimuli (Figure 6A). The neck connective response (measuring RMS area) to tactile stimuli was transient and corresponded to the onset and offset of the stimulus. The response to noxious stimuli was phasic-tonic encoding the entire duration of the noxious stimulus. Likewise, the neck connective response to continuous tactile stimuli (*n* = 12, means ± SEM: 0.16 ± 0.02) was brief and weak compared to that to the continuous noxious stimuli (*n* = 12, means ± SEM: 0.24 ± 0.03; Figure 6B). The averaged neck connective response to brief or continuous noxious stimuli was significantly higher than that of tactile stimuli (*P* < 0.05, Wilcoxon signed rank test, and *P* < 0.001, paired *t*-test, respectively).

### Involvement of the Head Ganglia in Nocifensive Behavior

**FIGURE 7:**
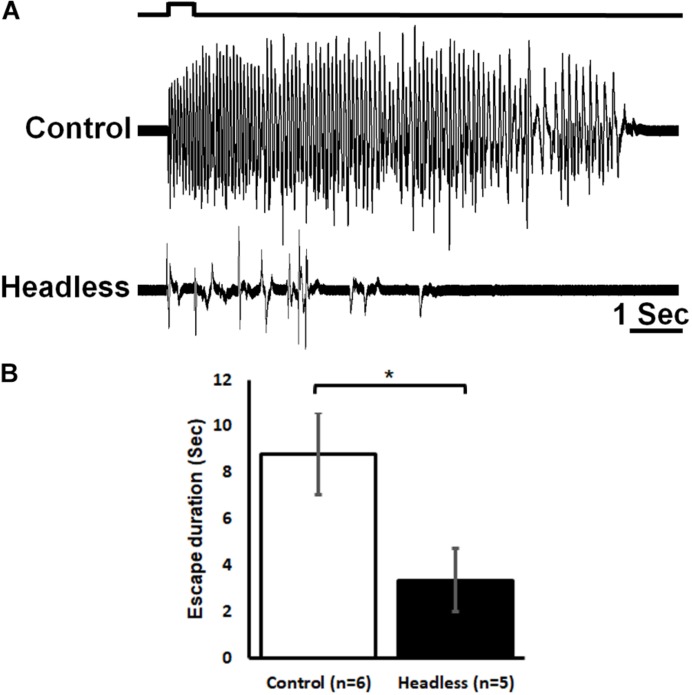
The head ganglia are required for proper escape response to noxious stimuli. **(A)** Response of control (upper trace) and headless (lower trace) tethered cockroaches to brief (0.5 s) noxious stimuli. **(B)** The escape response to noxious stimuli of headless cockroaches is reduced as compared to control cockroaches. Bars represent means ± SEM, significance is indicated with asterisk.

Given the transfer of nociceptive information to the head ganglia as demonstrated in the previous experiment, one could hypothesize that a proper nocifensive response to noxious stimuli in cockroaches may require descending information from the head ganglia (Figure 7). In this experimental procedure, we first applied a 0.5 s brief noxious stimulus to an intact cockroach and measured its escape movements. Such a stimulus recruited both tactile and nociceptive interneurons (Figure 6A) and elicited a strong startle-escape run in the intact cockroach (Figure 2). After removing the head, cockroaches (*n* = 5, 21 trials) displayed a shorter (means ± SEM in seconds: 3.4 ± 1.4, *P* < 0.05, *t*-test) nocifensive response to that of control cockroaches (*n* = 6, 28 trials. means ± SEM in seconds: 8.8 ± 1.8). But since tactile stimuli alone are known to trigger such a robust escape (Schaefer and Ritzmann, 2001), we cannot infer whether this escape response is due to the tactile component or the noxious component of the stimulus or a combination of both. Yet, if tactile stimuli are known to fail to evoke a full escape run in headless cockroaches (Schaefer and Ritzmann, 2001; Gal and Libersat, 2006), we show here that the noxious component of our stimulus is unsuccessful as well.

## Discussion

The essential ability of any animal to sense harmful stimuli with the potential of causing injury, and to perform a proper defensive behavior, is un-surprisingly evident also in cockroaches. By means of behavioral tests we determined that the response to pure noxious stimuli induces a more robust and longer lasting escape response as compared to other stimuli (Figure 2). The differences in escape behavior suggests the existence of a pathway for sensing and interpreting nociceptive information distinct from other sensory pathways.

In the present study, we confirm the existence of such a pathway by showing first the recruitment of noxious-sensing neurons in abdominal nerve 2 of the cockroach (Figure 3). When a tactile stimulus is applied to the cuticle, a transient (phasic) activity occurs at the onset and offset of the stimulus. When a noxious stimulus is applied, a prolonged (phasic-tonic) activity occurs and the units (sensory neurons’ axons) recruited are different from those that are activated by tactile stimuli (Figure 3A). This shows the existence of a labeled line entry for nociceptive information into the cockroach nerve cord. Although we do not know the source of the tactile evoked sensory response, we speculate it may arise from at least two potential sources. The first is campaniform sensilla in the dorsal cuticle. On each of the abdominal segments from 1 through 9, there are two campaniform sensilla on the dorsal surface, one near each lateral edge (Diwaker, 1973). The other is the lateral phasic receptors described by Florentine (1967). Although we do not present any anatomical data on the sensory neurons associated with the transduction of noxious stimuli in cockroaches, it is fair to assume that they should be similar to those identified as insect dendritic arborization (da) neurons in *M. sexta* (Grueber et al., 2001) and Drosophila (Grueber et al., 2002). Roughly the same numbers of such sensory neurons are found in Manduca and Drosophila with similar axonal projections in both insects’ species. Multi-dendritic sensory neurons resemble in their anatomy to the vertebrate nociceptive neurons with multiply-branched naked nerve endings attached with epidermal cells under the cuticle (Tracey et al., 2003). When testing Drosophila larvae with genetically silenced multi-dendritic sensory neurons, they are completely insensitive to noxious stimulation and fail to produce the nocifensive response (Hwang et al., 2007). The same type of sensory neurons is most likely associated with the transduction of noxious stimuli in cockroaches. Unlike mollusks, arthropods are covered with a hard integument or exoskeleton of chitin and hence, a comparison of insect nociception with that of other arthropods is demanded. With regard to crustaceans, crayfish respond with a nocifensive behaviors to noxious high temperatures. Antennal sensory neurons can detect short, transient high temperature stimuli, which is consistent with the antenna containing thermo-nociceptive sensory neurons (Puri and Faulkes, 2015).

In the cockroach central nervous system, different post-synaptic projection interneurons are recruited in response to tactile or noxious stimuli (Figures 4A,B). The response to tactile stimulus is accompanied with large amplitude spikes, occurring at the beginning and the end of the stimulus while the response to a noxious stimulus is accompanied with smaller spikes which occur throughout the stimulus. The existence of nociceptive interneurons in Drosophila and *M. sexta* nerve cord also supports the possibility of a pathway dedicated to nociception in insects (Ohyama et al., 2015; Hu et al., 2017; Tabuena et al., 2017; Yoshino et al., 2017). The difference in post-synaptic projection interneurons recruitment is also expressed in higher activity of the leg muscle EMG (Figures 4A,C). This higher activity indicates that the resulting behavioral response of the cockroach would be escape like behavior. To distinguish nociceptive post-synaptic projection interneurons from other known interneurons in the cockroach nerve cord the conduction velocity of interneurons was measured (Figure 5). The most studied interneurons of the cockroach nerve cord are the wind-sensitive ventral and dorsal giant interneurons (vGIs and dGIs) which have known velocities of 6–7 m/s for vGIs and velocities of 4–5 m/s for dGIs (Roeder, 1948; Spira et al., 1969; Westin et al., 1977). The response to wind stimuli starts with a brief high frequency burst in the vGIs followed by a longer burst of the dGIs (Roeder, 1948; Westin et al., 1977). The vGIs spikes have the largest amplitude due to the large diameters of the vGIs axons in the cockroach nerve cord (Spira et al., 1969). Conversely, dGIs have smaller diameter axons and consequently smaller spike amplitudes. Hence, spike amplitude and response latency are a reliable criterion to discriminate between these two sub-populations. The conduction velocities of the vGIs and dGIs measured in this work are similar to the known values published previously (6.7 m/s for vGIs and 4.7 m/s for dGIs) and are two to three fold faster than that of the measured nociceptive projection interneuron axons (3.7 m/s for large nociceptive interneurons and 2.7 m/s for small nociceptive interneurons). The lower conduction velocity of nociceptive interneurons and the smaller spike size of these interneurons (Figure 5) indicate that nociceptive interneurons have small diameter axons as compared to other axons in the nerve cord.

We found that the nociceptive information is carried along the nerve cord to the head ganglia (Figure 6). The pattern of the ascending activity is similar to the pattern of activity in the abdomen in which the response to tactile stimuli is transient and corresponds to the onset and offset of the stimulus and the response to noxious stimuli is phasic and ongoing throughout the stimulus. We also observed some persistent firing after removal of the noxious stimulus (Figures 3A, 4A, 6). It is unlikely to be caused by sensitization as this persistent firing of noxious sensory and interneurons occurred already on the first trial. We can only speculate regarding this post-stimulus activity and suggest that, after removing the heated probe, there must be still some residual heat which continue to recruit nociceptive sensory neurons. Ascending activity in the neck connectives indicate that nociceptive information reaches the head ganglia for further processing in order to execute the proper response to avoid the noxious stimuli. In drosophila larvae, local nerve cord pathway and brain both contribute to nocifensive behavior (Ohyama et al., 2015). Nociceptive sensory information is processed locally in the nerve cord but also send to the brain. Both shorter (local) and the longer (distributed) pathways converge back on the same command-like neurons in motor nerve cord. In adult drosophila, Neurons in the fan shaped body, which is part of the central complex in the adult brain, plays a role in the regulation of nociceptive heat stimulus avoidance (Hu et al., 2018). Silencing these neurons leads to a reduction in heat stimulus avoidance and activating them consistently triggers avoidance. Hence, a specific brain region may take part in general nociceptive processing. If nociceptive information reaches the head ganglia in cockroaches, it may imply the involvement of such ganglia in the nocifensive behavior. It is already known that wind or tactile stimuli elicit strong startle followed by escape running in cockroaches. When decapitated, such stimuli evoke a startle response but not an escape run (Schaefer and Ritzmann, 2001). And indeed, removing the head ganglia results in a reduction of nocifensive escape behavior in response to noxious stimuli (Figure 7). While we know that headless cockroaches do not show tactile evoked full escape response, the noxious component of our tactile-noxious stimulus does not induce a full nocifensive escape behavior. This suggests that the head ganglia are involved in the integration and modulation of nociceptive information. A proper nocifensive response cannot be executed properly without descending information from the head ganglia. Our investigation indicates how brain and nerve cord pathways interact with each other to contribute to the selection of a brief motor response (startle) followed by a long motor sequence (escape) in response to noxious stimuli.

In the nerve cord, nociceptive terminals recruit a distinct population of projection nociceptive interneurons with conduction velocity slower than that of tactile and wind sensitive projection interneurons (Figure 5). The nociceptive information is transferred to the thoracic segments where it engages local thoracic reflexes but also all the way up to the head ganglia (Figure 6). Likewise, the nociceptive information is transferred to local and projections interneurons in the spinal cord in mammals. At the spinal level, nociceptive input may trigger an immediate, fast protective reflex. Projections interneurons send their axons via the spino-thalamic tract to the thalamus and after, to various cortical areas in the brain (Purves et al., 2004). The conduction velocities of such projection interneurons are not known. Hence, we can speculate that the slower conduction of nociceptive information compared to somatic mechanoreceptive information is due in large part to slower velocities of sensory afferents in mammals and slower velocities of projections post-synaptic interneurons in insects. Finally, but beyond the scope of the present study, modulation of the nociceptive input occurs via opiate receptors binding molecules in both mammals and insects. Specifically, many studies have explored the existence and possible role of an opioid system in insects (Stefano and Scharrer, 1981; Romeuf and Rémy, 1984; Ford et al., 1986; Duve and Thorpe, 1988; Stefano et al., 1990; Dyakonova et al., 1999; Dyakonova, 2001). One Conundrum though is that no genes encoding for opioid-like peptides of receptors have been found in the Drosophila genome (Kreienkamp et al., 2002). Yet, Opioid-like substances are known to have “anti-nociception”-like effects and to modulate the threshold for escape in insects (Stefano et al., 1990; Gritsai et al., 2000; Gritsai et al., 2009). In our laboratory, we have shown that opioid agonists led to an increased nocifensive startle threshold in cockroaches (Gavra and Libersat, 2011).

In mammals and insects, nociceptors bear some similarities in that (1) The transient receptor potential (TRP) family of receptor ion channels are the predominant sensors and transducers in nociceptive neurons’ membrane (Tracey et al., 2003; Zhong et al., 2012; Jardin et al., 2017) and (2) the “dendrites” that bear these channels are not associated with an accessory structure (Grueber et al., 2001, 2002; Tracey et al., 2003; Purves et al., 2004; Smith and Lewin, 2009). In Mammals, they are called free endings and in insects, multi-dendritic sensory neurons. Both are located close to the surface of the tegument (mammalian skin and insect cuticle). As in mammals, these multi-dendritic sensory neurons are equipped with the transduction machinery for acid, heat and mechanical noxious stimuli. While we did not measure the conduction velocities of the axons associated with noxious stimuli transduction in the present study, we can speculate that, unlike in mammals, the differences between tactile and noxious fibers should be minimal. This is based on our sensory nerve recording (Figure 3) of axons from mechanoreceptors and nociceptive receptors which show little difference in spike amplitude indicating similar axonal diameters, and consequently similar conduction velocities. In the perspective of evolution, comparisons across taxa as distant as mammals and insects highlight again the working of convergent evolution for the emergence of nociceptive function. Hence, studies of nociception in insects is valuable as they seem to share many basic features of nociception with mammals.

## Data Availability

The datasets analyzed for this study can be accessed upon request to SE.

## Ethics Statement

The experiments performed comply with Principles of Animal Care, NIH publication no. 86-23, revised in 1985, and with the current laws of the State of Israel.

## Author Contributions

Both authors conceptualized the subject, designed and analyzed the experiments, and wrote the manuscript. SE performed the experiments and prepared the figures.

## Conflict of Interest Statement

The authors declare that the research was conducted in the absence of any commercial or financial relationships that could be construed as a potential conflict of interest.
